# Causes of Death and Physicians’ Knowledge‐Producing Responsibility—How Expectations Shape Interest in ‘Finding Out What Happened’

**DOI:** 10.1111/1467-9566.70267

**Published:** 2026-10-01

**Authors:** Mikaela Sundberg, Hedvig Gröndal

**Affiliations:** ^1^ Department of Sociology and SCORE Stockholm University Stockholm Sweden; ^2^ Department of Animal Biosciences Swedish University of Agricultural Sciences Uppsala Sweden

**Keywords:** clinical knowledge, judgement, medicine, sociology, unexpected death

## Abstract

Clinical knowledge production is fundamentally centred on patient problems, improving the lives of patients. If patients die, physicians also need to administer death. What is their responsibility to generate knowledge about causes of death? Based on semi‐structured interviews with 35 physicians, we show what sources physicians draw on, what they consider sufficient for making statements about causes of death and when and on what grounds they consider autopsies needed. We broaden and generalise the question of how physicians report causes of death by viewing it as a case of knowledge‐producing responsibility, the judgement about when knowledge is considered sufficient. By building this discussion on notions of expected and unexpected death, we also add to the sociological literature on categories of death.

## 1 Introduction

The primary missions of most physicians are to diagnose, treat, cure or, at least, provide care to patients. Clinical knowledge production is fundamentally centred on patient problems, and knowledge production is oriented towards rendering the problems of specific patients actionable (Berg 1992). When patients die, the situation is fundamentally altered. On the one hand, knowledge production is no longer oriented to curing or prolonging the life of patients. The organising goal that structures medical decision‐making is thus no longer achievable (Borgstrom 2015). On the other hand, some knowledge about the (dead) patient is still relevant to produce, or at least communicate: Physicians need to administer death and fill out a cause of death certificate, stating what caused death. It is an obligation to fill in the cause of death certificate, but the question we explore is what obligation to knowledge about death this implies from the physicians' perspective. How much about a cause of death is necessary to know? Concretely, we ask: What sources do physicians draw on and what do they consider sufficient for making statements about causes of death?

From a societal and epidemiological perspective, the prevalence of fatal diseases is important to measure for understanding their trends and for evaluating and comparing preventative efforts and healthcare systems (World Health Organization [WHO] 2022). The World Health Organization (WHO) has promoted standardisation of health statistics to enable global comparisons, and all member states are obliged to use a similar certificate form to report causes of death (Moriyama et al. 2011, 18, 27–28). The uncertainties surrounding and poor quality of cause of death records have been a long‐lasting concern among those compiling and using cause of death statistics, however (see, e.g., Maudsley and Williams 1996; Sehdev and Hutchins 2001; Moriyama et al. 2011). Such accounts tend to neglect the social judgements and processes connected to cause of death certification (cf. Armstrong 2021), including the difficulties involved in translating phenomenologies of death into the classification systems used for statistical purposes (cf. Bowker and Star 1999, 102; Sundberg and Gröndal 2026). In other words, they tend to neglect the physicians' perspectives that we study in this article.

In the sociological literature, studies of physicians' knowledge‐making regarding cause of death are, with few exceptions, limited. McAllum et al. (2005, 679) conveyed how general practitioners in New Zealand expressed uncertainty about the cause of death, doing their ‘best guess’ and filling in the cause of death certificate with something that made sense to them. According to Prior and Bloor (1993, 366) (UK), practitioners are aware of the inaccuracy of their reporting but view the task as completing formalities, where accuracy is not the aim. In contrast, the meticulousness of forensic pathologists in their death investigation is well‐documented by detailed studies (e.g., Timmermans 2006; Sundberg 2025; Sundberg and Sundberg 2026). Yet, forensic pathologists are atypical physicians. Their concern is never to treat and cure but only to understand death as a fait accompli. In this article, we delimit our analysis to the perspectives of physicians who fill in cause of death certificates of their former patients. This means that conceptions of death prior to the event may play a role.

The sociological literature on death and dying presents distinctive ways of defining categories of death. Prior (1989) discussed death as constituted by discourse, focussing on the category of unnatural death as articulated and performed in legal, political and clinical contexts, whereas Sudnow (1967) studied categories of death as socially organised, constituted by the activities of hospital personnel. Timmermans (2005), in turn, suggested how providers of healthcare broke ideals of ‘natural death’ and ‘good death’ to render individual deaths culturally meaningful. Others have shown how ideals of ‘good death’ vary across groups (e.g., Cain and McCleskey 2019; Seymour et al. 2007). Adding to this literature, we discuss categories of death based on physicians' reasoning about the determination of cause of death, shifting the focus to what happens after, rather than before, death. Specifically, we show how reporting physicians understand expected and unexpected death, which, in turn, shape and justify their, often limited, interest in understanding what caused death. We conceptualise this interest as reflecting the physicians' perspective on knowledge‐producing responsibility concerning cause of death.

### 1.1 Knowledge‐Producing Responsibility

What and how much do we need to know about something? The questions in this article relate to general issues regarding knowledge and responsibility. Within a philosophical context, the concept of epistemic responsibility is well known. Epistemic responsibility refers to an obligation to ‘know well’ and the cognitive activity of the knowers and would‐be knowers relative to that (Code 2020). It is thus based on an assumption that it is possible to know more or less (Code 2020, 11). According to Code (2020, 51), ‘evaluation of human knowledge‐seeking in terms of responsibility is instructive precisely because of the active, creative nature of that endeavour.’ Code (2020) uses knowledge and understanding interchangeably and relates responsibility to the cognition of the individual knower. Although Code (2020, 60, 62) does not argue for knowledge‐seeking as an individualistic endeavour but sees the cognitive authority of others and the epistemic community as imposing standards of achievement, their treatise remains within the philosophical realm, without considering social practices.

The concept of knowledge‐producing responsibility (Sundberg and Gröndal 2026) differs from Code's concept by drawing attention to the generation of knowledge and the activities that it entails, building on a key finding in the sociology of science and science and technology studies literature: The end of scientific fact‐making is never given or absolute but determined by social circumstances (Collins 1992; Galison 1987; Pinch and Bijker 1984). Knowledge‐producing responsibility extrapolates the question of where to stop to a wider set of professional work. This is relevant because many professional investigations entail instances of knowledge‐producing activities, even if they are comparably much narrower and specific endeavours compared with open‐ended scientific processes (see also Cole 2013). Consider, for example, pre‐trial investigations, where the police look for evidence and need to set priorities about what and how much to do (see, e.g., Liljegren et al. 2021), forensic cause of death investigations, where decisions about the scope of the investigation must take place (see, e.g., Hanzlick 2006; Sundberg and Sundberg 2026), and also clinical practice, oriented to knowledge about specific patients (cf. Pols 2012).

As a form of prospective, forward‐looking responsibility (cf. Cane 2002), knowledge‐producing responsibility pertains to expectations on knowledge connected to those who are responsible for producing knowledge but also to who they are producing knowledge for. In the professional realm, knowledge‐producing responsibility can be persistently attached to a specific profession, debated or shifting over time. For example, in the United States, forensic pathologists have gradually become more influential in showing the need for medical knowledge in coronial investigations (Johnson‐McGrath 1995). At the same time, the enduring mix of systems for investigating suspicious death across different states (see, e.g., Hanzlick 2006) indicates continuous struggle for authority over death investigations, and the knowledge production taking place within them. The question of who to produce knowledge for is reminiscent of the professional's orientation of service to others (cf. Goode 1961); however, knowledge can also be created for oneself, as well as for colleagues, the profession and/or other audiences. Knowledge‐producing responsibility is thus a way to raise and conceptualise questions related to how professionals perceive, relate to and give priority to these expectations, which can be more or less explicit or unanimous.

The central question that knowledge‐producing responsibility raises is: What is sufficient knowledge for the producers and for those who they produce knowledge for? Knowledge‐producing responsibility refers to the judgement about when to stop looking for or reading background information, collecting material, analysing or, in short, whatever knowledge‐producing activities one engages in. Such judgement relates to expectations and considerations regarding sufficiency in terms of scope, detail, depth and precision and to the professional discretion related to decision‐making based on that judgement. The concept thus shares with Code (2020, 11) the assumption that it is possible to know more or less but differs by its focus on knowledge production in relation to knowing enough.

The knowledge‐generating activities concerning cause of death that are open to general physicians and hospital doctors who fill in cause of death certificates are few. Nevertheless, they must make choices about what to do (search for) prior to making a statement about cause of death. This article explores what they consider necessary or sufficient to find out, combining the aspect of what the responsibility to know is (or what it is acceptable to be ignorant of)—epistemic responsibility in Code's (2020) sense—with the question of what is considered needed and relevant to generate—knowledge‐producing responsibility in our sense.

### 1.2 Background

We study cause‐of‐death knowledge in Sweden. About 95,000 people die per year in Sweden, and about 39% of those do so at hospitals, 38% in nursing homes, 20% die at home and about 2% elsewhere (2019; see Larsdotter et al. 2024). For every death, a physician must fill in a cause of death certificate and send it to the National Board of Health and Welfare for registration and subsequent statistical compilation. The certificate is based on the regulations connected to the International Classification of Diseases (ICD) and related health problems. The certificate includes boxes for filling in, on the one hand, a chain of causes from terminal to underlying causes and, on the other hand, contributing causes that are separate from the chain. For national statistical purposes, the underlying condition is most important and is defined by the ICD as ‘the condition that began the chain of events that ultimately led to the person's death’. However, this is not necessarily the way physicians understand or talk about death. In the remainder of the article, we focus on the descriptions and terms physicians use to talk about death, regardless of how they fit with ICD conceptions.

In Sweden, the type of physician that fills in the certificate depends on where death takes place and the circumstances of death. Physicians who establish death are obliged to contact the police if external factors (i.e., homicide, suicide, accident or malpractice in health) cannot be excluded as having led to death (Autopsy Act 1995, 832). The police then decide whether to request a forensic post mortem examination. Forensic pathologists at the National Board of Forensic Medicine conduct forensic post mortem examinations for about 6% of all cases of death per year, being responsible for certifying the cause of death for the cases examined. As clarified in the introduction, this work is beyond the scope of this article, which is concerned with all other cases, that is, when a general practitioner or hospital doctor assigns death. For these cases, it is up to the physician responsible for reporting the cause of death to decide whether material is sufficient, or if there is a need to request a so‐called clinical autopsy, and then fill in the certificate based on its results. Unless it is of ‘specific importance that cause of death is determined’ (Autopsy Act 1995, 832 9 §) or the dead person has communicated acceptance to post mortem examination (HSL 2 c §), clinical autopsies must obtain consent from relatives. If there are reasons for a forensic autopsy, a clinical autopsy should not be conducted (Socialstyrelsen 1996). Clinical pathologists perform autopsies in many hospitals in Sweden, and requesting physicians may attend if they so wish.

Following an international trend, clinical autopsies in Sweden have decreased. In 1969, clinical autopsies were conducted on 39% of all deceased (Rosendahl et al. 2021, 4); in 2024, less than 3% (Socialstyrelsen 2025). The physician requests the clinical autopsy in 43% of cases, 45% are requested in dialogue with relatives and, in 11%, relatives initiate the request (Rosendahl 2019, 24). Autopsy rates are higher for younger people than for the elderly, higher for those found dead at home than for death occurring at hospitals and higher for those at hospitals than for those in nursing homes (Rosendahl et al. 2021). Research reporting and discussing declining autopsy rates typically argue that the decline is problematic, because of the presumed value of post‐mortem examinations for improving the accuracy of cause of death certificates (e.g., Rosendahl et al. 2021; van den Tweel and Wittekind 2016). In this literature, the pertinent question is why clinicians no longer request clinical autopsies. Suggested reasons for the trend include physicians' lack of understanding of the value of autopsies for cause of death determination and quality control, technological advancements that have improved diagnostics prior to death and refusal to grant permission by relatives (e.g., Bayer‐Garner et al. 2002; Burton and Underwood 2007; Blokker et al. 2016; Hooper and Geller 2007).

How physicians deal with death and reason around the need for a clinical autopsy can be seen as an expression of their perspective on their knowledge‐producing responsibility—the need to generate knowledge about something, relative to whom and to what degree—regarding causes of death. Studying this complements the quantitative knowledge on clinical autopsy requests and suggested reasons why they are no longer relevant.

## 2 Methods, Data and Analysis

The primary material for this article consists of qualitative, semi‐structured interviews with 35 physicians, 15 general practitioners (GPs) and 20 hospital doctors, responsible for filling out cause of death certificates.

Recruiting GPs was difficult at first. In total, 23 managers of healthcare centres did not reply to our e‐mail request for interviews, and 15 physicians did not reply to direct requests. Informal channels turned out crucial (see also Asch et al. 2000; Petersson and Backman 2021). We recruited seven GPs through personal contacts achieved through the second author's prior research experience, five GPs through their healthcare manager, two via recommendations from previous interviewees and one from a research centre. Recruitment difficulties are also the reason why our material includes two retired, but still working, specialists. At the same time, we wanted variation in experience (interviewing both residents and specialists), geographic location (GPs working in cities and in the countryside, meaning different distances to most patients and to hospitals) and work conditions (working in healthcare centres and mobile teams). See Table 1 for specification.

**TABLE 1:** Interviewed general practitioners (GPs).

Physician	Geographic location	Work condition	Experience
GP1	City 1	Healthcare centre	Specialist (retired, still working)
GP2	Small town	Healthcare centre	Specialist
GP3	City 2	Healthcare centre	Specialist
GP4	Countryside	Healthcare centre	Resident
GP5	City 2	Healthcare centre	Resident
GP6	City 2	Healthcare centre	Resident
GP7	City 3	Healthcare centre	Specialist (retired, still working)
GP8	Countryside	Healthcare centre	Specialist
GP9	Countryside	Healthcare centre	Specialist
GP10	City 2	Healthcare centre	Specialist
GP11	Countryside	Healthcare centre	Specialist
GP12	City 1	Healthcare centre	Specialist
GP13	City 1	Healthcare centre	Resident
GP14	City 1/countryside	Mobile unit	Specialist
GP15	City 4	Mobile unit	Specialist

Our interviews were semi‐structured, including questions on dealing with death, the basis for and experiences of filling out the cause of death certificate, contact with the police and requests for clinical autopsy, striving for as many concrete examples as possible. The interviews lasted between about 45 and 75 min, on average about an hour. Except for one interview conducted using a video conference tool, all interviews were conducted in person by the second author.

For interviews about death occurring at hospitals, we chose a variety of specialities based on previous research on variation in autopsy requests, informal conversations with physicians working in different areas and the initial interviews with GPs. See Table 2 for specification. Based on our prior experiences of recruiting GPs, we made immediate use of informal contacts and recruited ten interviewees. The remaining physicians were recruited through research centres (2), healthcare management (2), directly through earlier interviews (3) and indirectly through earlier interviews (3). Our interview questions were similar to those we asked GPs. Because the distinction between expected and unexpected death had struck our attention, we also added an explicit question on the understanding of it, asked towards the end of the interviews with hospital doctors. The interviews lasted between about 45 and 90 min, on average about an hour long. Eight of the interviews were conducted using video conference tools, the others in person.

**TABLE 2:** Interviewed hospital physicians.

Physician	Speciality
HP1	Kidney
HP2	Infection
HP3	Internal medicine
HP4	Internal medicine
HP5	Surgery
HP6	Lungs
HP7	Surgery
HP8	Lungs
HP9	Oncology
HP10	Geriatrics
HP11	Geriatrics
HP12	Infection
HP13	Palliative medicine
HP14	Kidney
HP15	Palliative medicine
HP16	Kidney
HP17	Haematology
HP18	Haematology
HP19	Thorax surgery
HP20	Thorax surgery

All interviews were transcribed verbatim and sorted according to detailed coding schemes, using N‐VIVO. Coding of the material on GPs and hospital doctors was somewhat similar, with minor variation to capture specific characteristics of work at healthcare centres compared with that in hospitals. For the present analysis, we have used the material in codes about basis for assessment, clinical autopsy (reasons for and against the necessity of clinical autopsy), and for GPs, also on contact with the police. We have cross‐examined these codes in relation to codes including material mentioning ‘expected’ and ‘unexpected’ death. Working in‐depth with the material in these latter codes, we outline in more detail the meaning of these commonly used categories of death, which physicians refer to in relation to how much is necessary to find out about what caused death and for whom one is doing so, that is, the way we study knowledge‐producing responsibility in this article. Based on our material, we cannot infer the status of the categories expected and unexpected at the level of activities prior to death, but they are clearly important for physicians' sense‐making about death retrospectively. In addition, the article does not pertain to physicians' decision‐making about examinations, tests and treatment for living patients. We nevertheless draw upon talk about decisions for understanding the meaning of expected death. For the purpose of triangulation, we use interviews with eight clinical pathologists, working at four different clinical pathology units, as secondary material.

According to Swedish law (Lag [2003:460] om etikprövning av forskning som avser människor), no application for ethical approval is required for the research conducted within the research project, because it does not interrogate ‘sensitive areas’, as defined by the law. Although personal health is one such area, this applies only to the living. Consequently, information about previous health issues on dead persons does not require ethical permission. Advice from the university‐level legal counselling agrees with our interpretation that the research project does not require ethical approval.

To protect the anonymity of cited interlocutors, we refer to them as GPs (e.g., GP3) or hospital physicians (e.g., HP2), including their speciality. We have edited details of the cases referred to and translated all quotes from Swedish.

## 3 Findings

We present our findings in three main sections. After presenting the commonly used sources for assessing cause of death, we analyse in more detail the meaning of expected death, as a crucial aspect for understanding the importance, or rather lack of importance, physicians place on understanding cause of death. In contrast, when death is unexpected, physicians find it necessary to find out what happened and assume a greater concern among relatives, for whom they also wish to provide some knowledge. In consequence, it is more common to request clinical autopsies for such cases. Such requests may also be used as an attempt to redirect the question about cause of death to forensic medicine, via clinical pathology. In combination, these sections show how the knowledge‐producing responsibility—in terms of for whom knowledge is considered and assumed important to generate and how much that is necessary to find out—regarding cause of death is closely related to whether physicians conceptualise death as expected or unexpected.

### 3.1 Basis for Assessment

What sources do physicians turn to for filling out the cause of death certificate? In cases when physicians know the patient well, by having been responsible for treatment or care for a long time, they fill out the certificate on the spot. More commonly, they consult available information in the medical record. At nursing homes, the elderly residents, in need of frequent daily care, typically have a great deal of notes and many diagnoses and diseases listed in their medical record, and the responsible GP may know the patients by having rounded there many times. In consequence, GPs responsible for healthcare there have extensive background when assessing the cause of death. This does not mean that they are certain about their conclusions. Claiming to pick the ‘most plausible’ among the diagnoses, GPs talk a great deal about cause of death certification as ‘guessing’ (see also McAllum et al. 2005). A ‘guess’ is accepted, as one GP claimed, explaining that ‘if we have good information about co‐morbidity prior to death, then it's actually not very interesting to know the absolute terminal cause of death’ (GP9). Existing diagnoses are considered sufficient. However, information about recent development and clinical signs can be helpful for ordering diseases or conditions in the chain of events that the cause of death certificate requests. For elderly living at home, domestic health service may have noted something regarding the recent health condition. Although specific information may be limited and there is only a sense of ‘general fading away’, diagnoses noted in the medical records are typically considered sufficient for filling in the form for such cases.

Death at hospital generally means that recent notes and test reports are more specific and extensive compared with death at a nursing home (or elsewhere). Even in cases when death occurs rapidly after arrival to an emergency unit, examinations and test results inform about the immediate or short‐term condition. One kidney specialist described: ‘Even if someone arrives [to the emergency], doing really, really bad (…) we take an abundance of tests. We try to stabilise them and before we take them to the intensive care unit. On the way there, we do a computed tomography scanning, more or less. We do a great deal of things on very short time’ (HP16). A longer period of observation provides a better sense of the course of events, as one internist explained.

If they’re on the ward, you usually have many hours, days… sometimes you have weeks in a course of events that, well, someone arrives with an infection and they improve, then they have a new infection and then one tries to, then you can’t reverse it and then finally, no, this isn’t working. One has had such a long time following them. (…) It’s not that many surprises. You’ve kind of turned all stones that you think is of interest. You’ve had lots of time to take all blood samples and radiology examinations, or talked to other colleagues who are specialised in something else. (…) They are in all relevant senses demystified.

(HP4)

Two points are important here. First, having looked at everything ‘interesting’ and ‘demystified’ the patient prior to death, using medical technology, an autopsy would appear superfluous, reminiscent of arguments that clinical autopsies have declined due to improved technologies for diagnostics. Second, we learn from this quote how observation time is a factor affecting knowledge about the process leading to death. The intensity of investigations and treatment is another. Being in an intensive care unit means not only being under intense treatment but also under extensive surveillance. A surgeon related what could happen if there is some ‘trouble’ after a major surgery: ‘The patient is often connected both with ECG, monitoring of blood pressure, testing, perhaps not one but several times a day. Those courses of events that happen and that perhaps lead to death, they are pretty well known then’ (HP5). In sum, notes and diagnoses in the medical record, recent tests and investigations and direct observations are used and typically considered sufficient for reporting what caused death.

### 3.2 Expected Death

Knowledge‐producing responsibility related to finding out what caused death depends on whether physicians regarded death as expected or unexpected. This distinction is crucial, but what is understood as expected death is a ‘grey zone’ with multiple meanings and gradients. Swedish healthcare guidelines include some formal definitions of actions related to expected death. One is the decision about shifting from active treatment to so‐called end‐of‐life care (Socialstyrelsen 2025). Another is the so‐called breakpoint conversation (brytpunktssamtal), defined by the National Board of Health and Welfare (Socialstyrelsen 2013) as ‘a conversation when physician and patient discuss the stand to shift to palliative care in end of life’. An evaluation of Swedish palliative care showed that only half of the patients had such a conversation with a physician (Socialstyrelsen 2016) and extensive research demonstrates physicians' hesitance and uncertainty regarding communication about prognosis on death (see, e.g., Tate 2022 and references therein). In our interviews, physicians speak of having expected death but seemingly without having decided to end treatment or communicated their expectation to the patient. In practice, it is also blurry what such a conversation with a patient entails. For example, one GP said, ‘you can have different kinds of end‐of‐life care conversations’ (GP2). In the following, we explore the interviewed physicians' understanding and reasoning on the meaning of expected death (regardless of how these align with or deviate from formal definitions and healthcare recommendations) and its implications for what knowledge about cause of death that they find necessary.

#### 3.2.1 Prognosticated Terminal Illness

Decisions about end‐of‐life care or breakpoint conversations are definitive signs that death is expected. However, such decisions or conversations do not necessarily mean that death is expected to happen soon. Elder (2026) discusses the clinical ambiguity involved in decision‐making when there is certainty that the patient will die but when multiple treatment options are still available. We ask a related but somewhat different question: What happens in between prognostication of terminal illness and death in terms of knowledge production about how the disease develops or about if and how other complications arise and develop? The following quote provides an example:

You may have an end‐of‐life care conversation and then it’s someone who’s expected to die within a month or two, and then you get severe pain that a surgical measure could make better… then you can make such an examination or arrangement, perhaps put in (….) drainage and those things in the stomach, because one doesn’t want them to be in pain. The goal is always to take care of pain, anxiety, worry… that part, and sometimes it requires an examination. (…) There are many such things that can be palliative measures, but no investigation of causes of disease and such things.

(HP10)

According to the specialist in geriatrics cited earlier, evident suffering, related to complications, is taken care of. The point here is that there is no or very limited ‘investigation’. No attempts are made to monitor the development of illness or if some new disease has evolved, potentially affecting when or how death actually occurred. This is particularly the case with diagnosed cancer, where the trajectory is supposedly clear, as we see in this quote from a GP: ‘If you have someone with a cancer and then who's weakening and weakening and then they find him dead. What does it matter if there was a small myocardial infarction? Because you still consider it as that they have died from their cancer’ (GP11). One oncologist explained in more detail:

Our patients die, they have that severe diseases (…) We don’t care that much about the cause of death, we know that this patient, it’s going down (…) we don’t look, but we only focus on symptom relief, that it should be good and nice. The cause, the final cause of death, we don’t take any, when we have decided to lay down the weapons, there’s no testing, we don’t measure anything, no radiology or anything. It’s expected and it’s very unclear really, the basic cause of death.

(HP9)

When the trajectory towards death is prognosticated and the fight against the disease is over, cause of death is taken for granted. Knowledge about what happened between ‘laying down the weapons’ and death is limited, because the knowledge‐producing responsibility regarding cause of death is already fulfilled, as it were. The case is different prior to such prognosis and related decisions on cutting treatment. The oncologist went on

If someone arrives with a metastatic disease, a cancer patient for whom we have not decided about if there will be no measures, meaning resuscitation and intensive care and all that, then we go pretty far to see if it’s something reversible or not. In those cases [if they die] then we know more what it’s about, if it’s a bleeding or an infection.

(HP9)

Examinations close to death thus mean greater knowledge and understanding about what led to death. Yet, examination and treatment continue only as long as death is considered (temporarily) avoidable and stop if death is no longer considered unavoidable. Reminiscent of the oncologist, a physician in palliative care reasoned in the following way:

Why should we look after them [parameters] when the patient is about to die? Yes, symptom relief, the patient has trouble breathing, but I don’t look after if they have a pulmonary embolism, if they are at hospice with one more year to live. That’s kind of uninteresting. Or, if the patient is suddenly unconscious, was it a stroke or…? I don’t know. (…) You have an acceptance of that the patient can die.

(HP15)

Acceptance of death means a conception of death as unavoidable. Finding out more about how or why it happened becomes ‘uninteresting’. This suggests that it is no longer considered necessary to investigate, but a matter of preference, related to what is ‘interesting’ (which it, in the aforementioned quote, was not). In other words, investigation would be knowledge production for the sake of knowledge, in contrast to knowledge production oriented towards rendering problems actionable.

#### 3.2.2 Old Age

Although it is not necessarily clear at what age a patient is ‘old’ or at what age death starts to be expected (see also Kaufman et al. 2004), death at ‘old age’ is expected (see also Sudnow 1967). A kidney specialist described the meaning of ‘expected’ as ‘very much of a sliding scale in some way. But, expected, simply being old makes death much more expected. And what is old? Everything beyond 70, or so, and then the more diseases the patient has, the more expected death is’ (HP14). Somewhat similarly, an infectious disease physician related that expected death, referring to ‘someone who's old, sick (…)many bodily functions are weak, you have less resources, and then you know that an infection can push them over the edge’ (HP12). In any case, death will occur at some point: ‘We're programed to live a certain number of years and then you can mess it up by having a great deal of diseases, but even if you are not the slightest ill you die sooner or later’ (GP11).

The question knowledge‐producing responsibility begs is to what extent the inevitable event of death at ‘old age’ needs to be explained, and by what. One GP criticised age as explanatory: ‘Before you said elderly frailty, but I don't think it's good enough. We know that many are active until 100 years old. So why should we say that a 90‐year‐old died of elderly frailty? You don't die of age. You die of something’ (GP7). ‘Elderly frailty’ is an old‐fashioned expression that was used in Swedish cause of death reporting prior to international standardisation, but which is incorrect to fill in as a cause of death in the cause of death certificate, according to ICD rules. This is certainly insufficient for making physicians explore the cause of death at ‘old age’, however, especially when multiple diseases are present. A specialist in infectious diseases described:

If someone is, pretty weak to start with and very multi morbid and perhaps you don’t really get what happened (…)it feels more expected and not so strange that the patient dies (…) It’s not like one would jump on an autopsy because you think there was some small myocardial infarction or perhaps some thrombus in the lung or so. (…) Perhaps it led to something, but you must die of something when you’re old, in some way.

(HP2)

Although the physicians' understanding of old age as an explanation for death differs, ‘old age’ makes death unsurprising. Death at ‘old age’ is therefore generally, but not always, considered superfluous to investigate further.

### 3.3 Unexpected Death and Reasons for Clinical Autopsy

Requesting a clinical autopsy implies that available sources provide insufficient knowledge about what caused death. There are both similarities and differences regarding the reasons why GPs and hospital doctors request autopsies. It is common to state that a clinical autopsy is necessary when the dead person is ‘young’ or when death is ‘unexpected’. We explore this further by presenting how physicians view clinical autopsies as necessary when the medical record does not provide sufficient clues (about what could have caused death), when they assume that relatives are eager to or should know (more), when physicians want to know what happened, for themselves in relation to future patients, or when GPs and the police disagree about the need for a forensic autopsy.

According to one GP, 'regardless of age, if someone dies abruptly and one doesn't have an idea, then it's difficult to fill in the cause of death certificate because you have nothing to base it on. And then you request an autopsy of course’ (GP11). One reason for having no idea and nothing to draw from is that the medical record is empty. Another GP described: ‘We've had those when there hasn't been a single note in 30 years, who wants to stay away and hates healthcare. Then you must do an autopsy. (…) Last time, it was someone 75 [years old], there's nothing in the entire Sweden, on the patient’ (GP15).^1^ These accounts present the clinical autopsy request as the evident choice; one ‘obviously’ needs or ‘must’ request an autopsy to be able to state anything on the cause of death certificate. In other cases, a combination of factors motivates a clinical autopsy.

A woman was unhealthy, but she was not more than almost 60 years old, she died at home abruptly, some medical cause. (…) Then it was obviously an autopsy. (…) We didn’t really know what she died of. Even if she had some vascular disease and diabetes, it felt like it was perhaps pretty important also for the relatives to know what she died of.

(GP11)

This quote from a GP illustrates how the sudden death of a person at home, despite known diseases, can motivate an autopsy, but it also brings attention to another question related to knowledge‐producing responsibility: Who is knowledge considered important for?

Despite the fact that none of the interviewed physicians mentioned clinical autopsy requests initiated by relatives, it is common to refer to the fact that the family or relatives ‘also’ want to know in cases when someone died young or death is considered unexpected (see also Latten et al. 2023, 870). One reason to mention relatives is that their consent is often required, but it also reflects assumptions and expectations about when relatives would like to know more and thus shows who to produce knowledge for. This is reminiscent of Timmermans’ (2005, 1003) claim that the guiding principle for forensic pathological investigation is a utilitarian altruism, providing knowledge to relatives who have lost someone. Yet, knowledge about the dead can also be a step towards knowledge of importance for relatives about themselves. If the physician suspects that the deceased suffered from a hereditary disease, it is good for relatives to find out and screen for it or take actions to deal with it. Consequently, in these cases, (more) knowledge is considered important for both the physician and for relatives. One infectious disease physician explained:

Unfortunately, there are diseases you can get that you can die very quickly from and that can be important for the remaining family members to know what has happened, and it can also be medically‐induced. Some diseases can be hereditary. (…)I actually think more about, kind of, not to fill in the certificate correctly, necessarily, even if this is also good, but rather that you, as a doctor, wants to know what one has seen, but also for the remaining family members to know.

(HP3)

A significant point in this quote is the separation between a ‘correct’ cause of death certification and (the more important) knowledge about what was observed. Consequently, there is a difference between requesting a clinical autopsy to have something to state on the cause of death certificate, on the one hand, and to know, on the other hand.

Compared with GPs, hospital doctors expressed more frequently a desire to ‘know’ or ‘understand’ ‘what happened’ during the process leading to death, regardless of whether the patient had a disease that would have been legitimate to report as cause of death. One motive relates to the prognosis, as the section on expected death revealed. When death is unexpected at the time it occurred, physicians want to know what happened.

A woman around 35‐years‐old who received chemotherapy for breast cancer, standard treatment, and then suddenly comes in with, for example, infection and then has a cardiac arrest. Why? How strange. Then you want to know. What has happened? Is it the chemotherapy that has affected the heart or is it a clot in the lung? (…) There you want to know if it is something drug induced and then also for the relatives, if there is something we had been able to avoid, if there was something we missed.

(HP9)

In this example, the oncologist talks about a ‘sudden’, ‘strange’ case of death, where finding out the reason for death is related to finding out if something could have been done differently, in other words, to avoid death. Occasionally, the reason for hospital doctors to request an autopsy is not so much to understand what happened to the specific person but to use understanding of the past to learn and avoid the death of future patients (e.g., understand treatment) (see also Christakis 1998, 79). It is hence a matter of generating actionable knowledge for treatment.

In our terminology of expected/unexpected death, there are also border cases when death is not expected, but the stakes are high. This is the case when physicians relate to the death of patients as avoidable, and to their treatment, such as advanced surgery, as necessary for survival, thus being a matter of ‘saving’ the patient. Consider thorax surgery: ‘Here almost all cases of death are unexpected (…) We have almost no one here who should die (…) We have chosen kind of, the little tip of the ice berg (…) those who you do surgery on are those who you really, who shouldn't die, who you should save’ (HP19). This quote from a thorax surgeon implies great responsibility for maintaining life, aligning with concern about understanding what happened in cases when death is nevertheless the outcome. Notably, clinical pathology interviews reveal that it is extremely rare for physicians to attend autopsies. Thorax surgeons appear as exceptions, as we see in this quote:

We almost always go and watch the autopsy. It’s not that often people die here (…) We are often very curious about how it looks and if there’s something we could have done different? How did it look? How did the anatomy actually look, where we couldn’t see? (…)There’s a great deal that we haven’t seen during surgery that we can get an answer to (…) or something one can learn from.

(HP19)

Thorax surgeons who perform advanced, high‐risk surgery may experience life and death as in their hands, and consequently, death during surgery as a sudden failure of the lifesaving effort. Considering the request for an autopsy, and the time taken to even go and watch it, suggests how responsibility for patient survival could be connected to responsibility for finding out more about how the patient nevertheless died. This is potentially actionable knowledge, of use when treating other patients. If death is considered inevitable, such knowledge is unobtainable. When death is expected, prognostication builds on assumptions about what will lead to death (i.e., its cause). This is the typical death faced by, for example, oncologists. According to Sudnow (1967, 92), death foreseen as a matter of fatal causes, which treatment cannot deal with, is the ideal form of dying from the physicians' perspective. Yet, the forms of dying that physicians meet depend on their speciality. The different interests in exploring the dead are likely connected to the working experiences that various medical specialisations entail when it comes to dealing with death and dying. Larger comparative samples than in the current study are necessary for establishing how.

#### 3.3.1 Shifting and Negotiating Knowledge‐Producing Responsibility

We have so far addressed clinical autopsy as a means to generate more knowledge and understanding for oneself as a physician and/or for relatives. Requesting a clinical autopsy can also be an attempt to shift knowledge‐producing responsibility elsewhere; to someone or somewhere else. In our interviews, this is exclusively revealed in GPs’ accounts about certifying death outside healthcare institutions.

Recapitulating from the background section, physicians are obliged to contact the police if they encounter cases of death where it cannot be excluded that unnatural (meaning caused by accident, suicide or homicide) courses of events led to death. When GPs enter scenes of death outside healthcare institutions (typically indoors), they observe whether there is ‘social misery’, ‘beer cans and bottles of booze’, ‘blister packages’ or other signs that the case should be taken care of by the police. GPs who testify death in private, individual homes commonly also claim to check that there is ‘no knife in the back’ and mention that they look for traces of violence or lesions. Despite the fact that physicians are required to examine the entire skin surface when testifying death, our interviews suggest that this is circumstantial, and in practice, rarely the case: None of the GPs reported undressing and examining the body unless something seemed obviously ‘weird’ or ‘suspicious’. However, signs are not always ‘obvious’. One GP said: ‘If you're in doubt, then it's clearly a case for the police (…) It's pretty convenient because then they will fix (…) [a] forensic medical investigation’ (GP7). The point is not to imply laziness among GPs but to illustrate that ‘doubt’ is enough for shifting the responsibility to forensic pathology. Yet, it is only the police who have the right to demand a forensic autopsy. What happens when physicians and police officers disagree about the need for it? The following quote from a GP working for a mobile unit—the type of GP facing death at home most frequently—provides an example:

At the end of the day, the police decide. So we can end up in a situation where we feel ‘this is an injury, an accident, you should take this’. ‘No’, says the police. ‘We don’t think so.’ (…) Then we almost always file a request for a clinical autopsy, in that request we write that we think this is likely a police case.

(GP14)

Another GP who worked for a mobile team spoke a great deal about contact with the police when something does ‘not feel right’.

Sometimes you get this kind of feeling that, what the heck, something isn’t right here. Then we’ve asked the police to come and have a look, and sometimes they say ‘no but this doesn’t feel like it’s something for us’ and you write that [in the autopsy request], and then it happens sometimes that the [clinical] pathologist says ‘but there’s something which isn’t right here, forensic medicine must take this’.

(GP15)

Both of these quotes illustrate negotiation between physicians and the police about who should take care of cases and how GPs occasionally request a clinical autopsy as a detour, hoping the case will be directed to forensic medicine via findings identified by a clinical pathologist. Such cases are occasionally transferred to a forensic medical unit for forensic examination, ordered by the police, but upon request (also) from the clinical pathologist. The knowledge‐producing responsibility has shifted.

## 4 Concluding Remarks

This article set out to study physicians' perspectives on their obligation to knowledge about cause of death, and more specifically, what sources they use and find sufficient for reporting a cause of death, depending on the cases of death they need to report. Viewing how physicians report causes of death as a case of knowledge‐producing responsibility situated these questions within a more general context and led us to explore who physicians produce knowledge about death for and when they consider more investigation (a clinical autopsy) necessary.

Our analysis adds to prior awareness of physicians' lack of interest in cause of death certification by providing a more systematic and detailed account of physicians' knowledge aims, thereby offering, first, a more nuanced view on cause of death reporting compared with studies from the perspective of users of statistics and second, a richer background to discussions on the current decline of clinical autopsies. More specifically, the analysis shows the significance of physicians' understanding of expected and unexpected death when it comes to their perspective on knowledge‐producing responsibility regarding cause of death. It is well known that age shapes whether patients receive lifesaving efforts (e.g., Sudnow 1967; Timmermans 1998). We show how limited interest in the causes of death appears to follow decisions to restrict investigation and treatment, based on prognostication of chances to survive. However, prognostication of death is difficult, sensitive and often wrong, with overestimated life expectancy being common (Christakis 1998). Christakis (1998, 235) argues that physicians feel obliged to think about what will happen and why when a patient exhibits ‘atypical’ features of a disease, meaning that the expected outcomes for the patient deviate from the usual outcome in such a group of patients. Our analysis shows a similar type of reasoning in the sense that unexpected death triggers questions about what caused it, whereas expected death does not. However, our material does not allow us to answer whether the categories of death referred to were in fact reasons affecting activities prior to death or retrospectively adjusted accounts casting death as more anticipated than it was (see also Christakis 1998, 28). (It is most likely a combination.)

Our interview material suggests that recent examinations provide physicians with material they consider sufficient for them to state something. Unless a patient's unexpected degeneration and death evoke the potential of providing lessons to be learnt for the future or because it is called for by relatives, exploring more is considered unnecessary. When medical records are empty of symptoms or diagnoses, a clinical autopsy is required to have something to write in the cause of death certificate, but not necessarily for understanding. Given our findings, it is thus unsurprising if (that) physicians do not request clinical autopsies for the sake of accuracy of cause‐of‐death reporting. Physicians sense a responsibility to generate cause‐of‐death knowledge for themselves as a specific type of physician, in charge of specific treatment, for specific diseases and for the relatives of a specific person. (In contrast with everyday clinical knowledge production, they are no longer oriented to the patient, if a dead person can be conceived of as such.) This empirical conclusion paves the way for a theoretical conclusion: The distinction physicians make between understanding what caused death and stating a cause of death relates to the difference between Code's epistemic responsibility and our definition of knowledge‐producing responsibility. This is because epistemic responsibility can be seen as judgement relative to personal knowledge, whereas knowledge‐producing responsibility also includes knowledge generated for others, where the communication of that knowledge inevitably comes to the fore.

We end with a note of practical importance. According to the National Agency of Health and Welfare (Socialstyrelsen 2022, 4), it is ‘unclear which examination routines the individual physicians use’ for the dead. Forensic pathologists in Sweden have argued for the risk that cases that would entitle forensic medical examination, thus potential suicides, accidents and homicides, are missed (Pettersson and Eriksson 2014; Wärn and Zilg 2019). Considering that 50% of all cases of death reported by the National Board of Forensic Medicine (Rättsmedicinalverket 2024) are classified as disease‐related (i.e., natural), it seems as if the bar for referral is low and the risk of missing suicides, accidents and homicides is not overwhelming. Yet, some of our findings are still noteworthy. Clinical autopsies serve as a means for GPs to deal with cases when there is disagreement with the police about whether criteria for forensic autopsies are fulfilled. Interviews also suggest that physicians' inspections of bodies and surroundings found at home are unsystematic. Taken together, this underlines the risk forensic pathologists in Sweden have pointed out earlier. Further research should study the negotiation between the police and physicians regarding the classification of and responsibility for taking care of (that is, produce knowledge about) the borderline cases.

## Author Contributions

**Mikaela Sundberg:** conceptualization, funding acquisition, writing – original draft, methodology, writing – review and editing. **Hedvig Gröndal:** investigation, methodology, writing – original draft.

## Funding

This study was supported by the FORTE, Swedish Research Council for Health, Working Life and Welfare, 2023‐00846.

## Ethics Statement

According to Swedish law (Lag [2003:460] om etikprövning av forskning som avser människor), no application for ethical approval is required for the research conducted within the research project. Projects on human subjects interrogating what is defined by the law as sensitive areas require ethical approval. Considering the focus on professional practices, perspectives, reasoning and judgements in the project, it does not interrogate what is defined as a sensitive area. Given the focus on perspectives in medical practice, it is worth pointing out that personal health is a sensitive research area according to the law. This applies only to the living, however, and not to the dead, where ethical restrictions pertain to the use of bodily material exclusively. Consequently, information about previous health issues on unidentified dead persons does not require ethical permission. The university legal counsellor on ethics, who has advised on this question, agrees with the interpretation that the research project did not require ethical approval.

## Consent

The authors have nothing to report.

## Conflicts of Interest

The authors declare no conflicts of interest.

## Permission to Reproduce Materials From Other Sources

The authors have nothing to report.

## Data Availability

The data are not publicly available due to privacy and ethical restrictions.

## Endnotes

^1^
The accessibility of various medical records, particularly for physicians working for mobile units, is uncertain. According to interviewed clinical pathologists, a great deal of autopsy requests come from such physicians.
